# Reducing Oral Health Disparities: A Focus on Social and Cultural Determinants

**DOI:** 10.1186/1472-6831-6-S1-S4

**Published:** 2006-06-15

**Authors:** Donald L Patrick, Rosanna Shuk Yin Lee, Michele Nucci, David Grembowski, Carol Zane Jolles, Peter Milgrom

**Affiliations:** 1 Department of Health Services, University of Washington, Seattle, WA, USA; 2 Deparment of Sociology, University of Washington, Seattle, WA, USA; 3 Northwest/Alaska Center to Reduce Oral Health Disparities and School of Dentistry, University of Washington, Seattle, WA, USA; 4 Department of Anthropology, University of Washington, Seattle, WA, USA

## Abstract

Oral health is essential to the general health and well-being of individuals and the population. Yet significant oral health disparities persist in the U.S. population because of a web of influences that include complex cultural and social processes that affect both oral health and access to effective dental health care.

This paper introduces an organizing framework for addressing oral health disparities. We present and discuss how the multiple influences on oral health and oral health disparities operate using this framework. Interventions targeted at different causal pathways bring new directions and implications for research and policy in reducing oral health disparities.

## Introduction

Reducing health disparities is a major goal for public and private health agencies in the United States [1] for health professionals and for the public at large. In 2000, the Surgeon General highlighted oral health as a major component of general health and well-being [2]. Oral health implies much more than healthy teeth. The mouth is both a cause and a reflection of individual and population health and well-being.

Persistent and consequential oral health disparities exist within the U.S. population, and reducing these oral health disparities is central to the overall goal of improving population health. What these disparities are, what causes them, and how to ameliorate and prevent them requires awareness, research, knowledge accumulation, and translation of this knowledge into action. Finally, reducing oral health disparities requires the ***will ***to act. Changes are needed in resource allocation, in social and public health policy, in community organization, in the provision of effective dental health care, and in professional and individual behavior.

Processes and mechanisms that may be called "determinants" operate at all levels of our society. Determinants of oral health disparities represent a complex mix of the biological, the behavioral, the cultural, the social, the economic, and the political.

We present a conceptual framework for investigating oral health disparities, with a focus on social and cultural determinants. Why a conceptual framework? This framework recognizes that inequality is produced through dynamic sociocultural processes. To reduce disparities, a cumulative science is needed that places knowledge gained from research, practice, and expert deliberation into a framework that identifies the modifiable mechanisms by which we can act to narrow inequalities. This paper focuses on dental caries (or tooth decay), because it is the major oral challenge U.S. society faces. The model, however, also applies to other oral health disparities, including periodontal diseases, oral pharyngeal cancer, and congenital and acquired facial differences.

## Definition: How Oral Health Disparities Work

Three key concepts are social determinants of health, health disparities, and population health. Understanding how these concepts apply to oral health requires definition and examples.

***Social determinants of health ***refer to both specific features of and pathways by which societal (including cultural) conditions affect health and well-being. Perhaps a misnomer but currently in widespread use, the notion of *determinants *suggests clear causal pathways that are often anything but clear. We concentrate on social and cultural conditions that might be altered by programs and policies. Examples are income, education, social capital, occupation, community structure, social support, availability of health services, and larger forces such as structural inequality, cultural beliefs and attitudes, and legal channels. Social determinants interact with biological and personal determinants at a collective level to shape individual biology, individual risk behaviors, environmental exposures, and access to resources that promote health. A graded relationship between social position and health status affects all persons in the social hierarchy [3].

Through the process of social stratification, individuals are divided into subgroups. They are differentiated based on attributes that are considered important by society, such as income, race, sex, and education. Once the process of differentiation has occurred, individuals are evaluated based on their attributes. Individuals with more favorable attributes are sorted into a higher social status in the hierarchy, which subsequently determines the provision of rewards. This social hierarchy provides differential benefits to individuals who occupy different positions. Hence, stratification functions as a process that formalizes inequality in the form of unequal access to valuable resources, such as quality housing, education, health care, and dental care. Thus, low-income groups, racial minorities, and people with disabilities are often found to have higher hospitalization rates and more emergency room visits than people of higher income, often due to the unhealthy and unsafe environments in which they live [3].

***Health disparities ***is a term that describes a disproportionate burden or risk of death, disease, disability, and ill health on a particular population or group. Healthy People 2010, a national health promotion and disease prevention initiative, placed primary concern on gender, race or ethnicity, education or income, disability, geographic location, and sexual orientation. Oral health disparities are often poorly understood. Women report more dental visits than men, yet few women receive dental care during pregnancy, a critical period [2]. Older individuals go to the dentist less frequently than the general population, are in poorer oral health, and have a lower quality of life as a result [4].

***Population health ***is a term that focuses on the interrelated conditions and factors that influence the health of populations over their life course, identifies systematic variations in their patterns of occurrence, and applies the resulting knowledge to develop and implement policies and actions to improve health and well-being. The goals of a population health approach to oral health are to maintain and improve the oral health of a given population and to reduce inequities in oral health. Methods for addressing disparities are emerging through social epidemiologic research and public health applications [5].

Health insurance inequalities contribute to oral health disparities [2]. In the U.S., access to oral health care is mediated through the availability of private dental insurance or poorly funded public oral care. Private dental insurance facilitates use of preventive oral care and mitigates negative effects of oral diseases. Public oral health care funds preventive care but few eligible persons actually receive it [2].

The RAND Health Insurance experiment provided evidence that providing free dental care can improve oral health in low-income preschool children [6]. Access to dental insurance, however, does not always guarantee reduction in oral health disparities [7]. In many American Indian and Alaska Native communities, oral health disparities exist even though dental care is theoretically available without cost as a function of tribal status. For tribal communities, the primary influences include a combination of factors, such as: geographic isolation, ethnic differences in social and cultural values, cultural and socio-economic changes that have strongly affected diet, lack of education as a consequence of social, political and cultural peripheralization at the hands of the dominant culture, and overall low levels of income that affect all of these factors.

The gap between available preventive oral technologies and their full dissemination and application also widens oral health disparities among different social and ethnic populations. Enormous growth in scientific technologies has led to remarkable public health advances in nutrition, immunization, personal hygiene, sanitation, and water and air safety. Yet, as a society, we continue to struggle with the challenge to allocate human and financial resources effectively to reduce social disparities in oral health. The "inverse care law" operates here. That is, those who most need care do not get it [8,9].

Tooth decay, which occurs chiefly during childhood and adolescence, is a transmissible bacterial infection. The major reservoir from which infants acquire tooth decay is through transmission of *mutans streptococci *from mothers and between infected and uninfected children [10]. Among some groups a child's extended family may very likely be responsible for significant transmission from adults to children. Efforts to prevent the transmission of bacterial agents begin with prenatal care and continue throughout the early childhood. Suppression of maternal bacterial reservoirs through preventive and curative treatment can prevent or delay the infection of young infants among certain affected groups [11,12].

Dental caries can be prevented or its impact lessened through lower consumption of sugar-rich foods and through water fluoridation, school- or home-based topical fluoride programs, supervised toothbrushing with fluoridated toothpaste at school, and toothpaste distribution schemes. For populations that are more severely impacted by tooth decay, additional measures, such as dental treatment or antimicrobial therapy, are required to control the disease [11,13].

Unfortunately, newly developed dental treatments and technological advancements that enhance prevention of tooth decay are unevenly distributed across segments of the population [14,15]. Differing values may deter the adoption of efficacious preventive behaviors. Poor teeth and poor oral health have different meanings to different segments of society, and values coupled with perceived available choices may influence what persons are willing to do to prevent oral problems. For example, some persons may believe that tooth loss is inevitable regardless of what they do. Others may adopt habits conducive to tooth preservation.

Research and development in the relatively small dental industrial sector worldwide is very limited. Even major multinational companies based in the U.S. invest little money in developing new preventive technologies to help ameliorate oral health disparities. So-called new technologies, such as fluoride varnish or Atraumatic Restorative Technique using glass ionomer, are either imported from Europe or created for use in developing countries. Federal device and drug regulations, administered by the U.S. Food and Drug Administration (FDA), are stringent in protecting the safety of the population and in evaluating efficacy. These regulations and the "market place" create economic disincentives to private enterprise for new research and development for preventive care. Little public subsidy exists for development of new preventive dental technology. Most promising devices, such as slow-release fluoride vehicles or better antimicrobials, sit unused on the lab shelf.

The complexity in addressing oral health through dental care can be illustrated by the experience in Head Start. Tooth decay attacks largely before children are enrolled in Head Start, and damage to the teeth is already done. At the same time, the children are too young to benefit from targeted approaches to prevention of dental caries in first permanent molars because the teeth have not yet erupted. School systems do not follow up on attempts during the Head Start years to arrest tooth decay, and children in these populations often go on to experience unchecked tooth decay in their permanent teeth.

## A Snapshot of Oral Health Care Disparities in the U.S

According to *Oral Health in America: A Report of the Surgeon General*, dental caries is the single most common chronic childhood disease in the United States [2]. Children living in poverty [16], including those with disabilities, are most likely to be affected by dental disease. Dental caries in young children is five times more common than asthma, and seven times more common than hay fever [1]. Disparities are also unequally distributed among adults. Low socioeconomic status, minority status, and unemployment are associated with patterns of infrequent preventive dental care and high rates of dental disease [17].

Lowering the cost of dental insurance has yielded little significant progress. Although the U.S. has made strides in lowering the cost of medical insurance via federal programs such as Medicaid and Medicare, low-income patients have reduced access to dental care because so many dentists refuse to participate in Medicaid and Medicare does not include dental coverage [18]. Gortmaker's (1981) examination of Medicaid utilization between 1973 and 1977 found that Medicaid increased utilization of dental service among children only during the early years of the research study [19]. Mueller (1984) examined utilization patterns of dental care among children with and without Medicaid and found that in 1977 the rate of utilization by children with Medicaid was 35 percent – only 10 percent higher than those without Medicaid – and still well below the national average of visits for children with private dental insurance [20].

Among older adults, dental health problems are cumulative and just as prevalent as in childhood. According to the U.S. Census Bureau, nearly 35 million Americans were 65 years or older in 2003. Oral diseases in this group affect older adults who are economically disadvantaged, lack dental health insurance, and are members of racial and ethnic minorities. Having a disability, being in an institution, or not being able to leave the house also increase the risk of poor oral health [2]. According to the 2003 Oral Health in America Report, "A State of Decay: The Oral Health of Older Americans," about 30 percent of adults 65 years or older no longer have any natural teeth, which is a condition that affects food intake and nutrition.

Dental benefit levels often increase only when state officials are sued by advocates for poor people. Head Start has attempted to increase access but, lacking the ability to provide direct services, is stymied in this effort by needing to use Medicaid as its payment system. As for older adults, Medicaid provides limited dental insurance coverage for maintenance of oral health for persons with very low incomes, but these programs inadequately address the needs of older adults with a disability, living without a primary caregiver, or in a nursing home.

Currently national statistics are not available to compare the utilization rates of dental care between children with and without Medicaid, or between older adults with Medicare and those without. Yet a recent cohort study that investigates the effects of WIC participation on children's use of oral health services in North Carolina showed that 53,591 of all 81,518 live births were enrolled in Medicaid, and about 12 percent stayed continuously enrolled during the first five years [21]. Children whose mothers participated in WIC for a full year were about 1.7 times more likely to have two or more dental visits per year than those children who never participated in WIC [21].

Numerous explanations have addressed the under-use of dental services offered through Medicaid, including explanations that involve the rejection of patients with Medicaid by dentists because of low and inconsistent reimbursement rates, frequently failed appointments, and a possible reluctance to treat patients whose dental problems may be complex and time consuming [22]. Related to this is the problem of recruiting dentists willing to serve populations living in remote rural areas or in urban areas that are considered undesirable [23].

Another major component in the underuse of available dental services is the culture gap between dentists and patients from varying social and ethnic classes [24]. Dentists and their patients differ in educational level, commitment to oral health values, and in the "culture" of dental practice [6]. Dentists respond to patients in different ways based on the patient's characteristics [25-29]. This cultural divide sometimes leads to discrimination. Treatment selection and quality of care also depend on the characteristics of the dentist-patient relationship [30].

Receptionists – the gatekeepers of the dental office – are also implicated in such discrimination. For example, Lam, Riedy, and Milgrom [31] found that staff members' personal connection to Medicaid-insured patients; staff members' attitudes about Medicaid-insured patients; and staff members' perceptions of Medicaid-insured patients' barriers to care affect Medicaid patients' willingness to seek dental care. An additional problem contributing to inequalities is the lack of preparation of dentists to treat children. Few dentists leave their training with as much skill and knowledge in treating children as they acquire in treating adults. The majority of dentists refuse to see preschoolers.

The nation's dental schools largely have failed to recognize their own role in reinforcing disparities. Increasingly the dental schools themselves reject Medicaid- covered patients, further institutionalizing this discrimination. Programs such as the widely cited ABCD Program in Washington State have attempted to remedy this component (that is, the problem with dental schools, or the underutilization problem?) of the disparity problem [32].

In addition to the difficulty of finding a dentist willing to take payment from Medicaid, patients with limited socioeconomic resources have to evaluate a whole web of factors associated with visiting a dentist; for example, the amount of time and money associated with one dental visit, the difficulty of finding reliable transportation, and the problem of taking time off work [33]. Therefore, eligibility for Medicaid insurance does not necessarily result in patients' enrollment, and enrollment does not ensure the availability, accessibility, and obtainment of needed dental care.

Patterns of under-use of dental care also emerge from cognitive stereotypes and discriminative practices of providers, patients, and the health-care system in which decisions about treatment are made [34]. Patient characteristics (e.g., race, ethnicity, age, income, gender, marital status, amount and type of health insurance, education level, cultural values associated with oral health, level of perceived disease burden, and disease severity) influence dental treatment and treatment decisions. Simultaneously, provider characteristics, such as practice specialty, practice style, attitudes, or bias about a patient's race, ethnicity, gender, class, and social background, may influence the type and quality of service provided [35,36].

A comprehensive review of empirical evidence has revealed that dental care, particularly care for patients from vulnerable groups, is a complex process. A whole range of factors contribute to under-use of Medicaid services. On the patient side, cultural values, education, prior experience with dentists, perceived value of dental care, and access issues influence care seeking. On the dental professional side, practitioners' peception of poor patients, financial costs, time, and reimbursement issues can influence delivery of oral care.

## Existing Models of Oral Health Behavior and Service Use

Although an extensive body of theoretical and empirical literature on oral health has been published, a comprehensive dynamic model explaining social disparities in oral health has yet to emerge [25,37-41]. A thorough review of all models goes beyond the scope of this paper. Therefore we discuss basic models of the use of dental health services. These models concentrate on *dental services *rather than *oral health disparities*.

Currently, our knowledge of dental care use is fragmented. The most commonly applied behavioral and social interaction models include Anderson's predisposing, enabling, need model of health services use [42]; Rosenstock's (1966) health belief model; Mechanic's (1978) factors of sociocultural/psychosocial model [43]; Antonovsky and Kats' (1970) preventive dental behavioral model [44]; Fishbein and Ajzen's (1975) model of belief, attitude, and intention [45]; Manning and Phelps' (1979) model of the demand for dental care [46]; and the social exchange model by Grembowski et al. [25].

Reviews of these models reveal several common themes [25,46-48].

First, most behavioral models assert that an individual's use of health services is a function of the perceived threat of disease, past use of medical services, and perceived value of action. In particular, the individual has to be psychologically ready to become aware of a symptom as a problem and must then choose to visit a health-care professional as the appropriate action. A variant of the main theme of the behavioral models is Manning and Phelps' demand model of dental care, which casts the demand of dental care in economic terms of "demand" and "supply." Within the context of household, dental care is a weighted outcome determined by income, price of treatment, time cost, variables affecting tastes and preferences for preventive care, agreement among members of the joint household with respect to altering market opportunities, and the amount of income forgone due to seeking treatment, in conjunction with the expected expenses of dental treatment.

Second, the individual must perceive the preventive measure/medical service as feasible and efficacious such that it would reduce the perceived severity of the dental condition and would outweigh the gains from the associated psychological, physiological, monetary, time, and/or other types of costs [40,49-52]. Alternatively, Antonovsky's salutogenic model emphasizes individual sense of coherence (comprehensibility) as the primary mobilizing resource through which patients utilize their ability to deal with stressful events (manageability), and to possess the motivation, desire, and commitment to cope (meaningfulness) [53].

Third, the probability of a dental-care episode depends on the social interactional exchange nature of the patient-dentist relationship. As a variant of the behavioral model, the social interaction model emphasizes the power dynamics among individual characteristics of the patient, the provider, and structural characteristics of the health-care provider. During an episode of dental care, the dentist occupies an authority position – the "gatekeeper," who controls the flow of information exchange (e.g., concern for the patient's general health) and the extent to which the patient is informed of her/his dental needs and treatment options [25,54].

Conversely, a patient may demand information regarding treatment options and remedies from a dentist and threaten to withdraw visits to that dentist. Similarly, dental health insurance providers, including government, may withdraw options for various dental treatments, based on the imbalance of economic costs and potential rewards for the insurance provider or because of perceived short-term savings to government. A very good example of this phenomenon was reduction of adult Medicaid dental benefits in Maryland, resulting in higher utilization of the hospital emergency room [55]. Hence, a dental visit, according to the social interactionist perspective, involves a reciprocal, dynamic flow of power among the dentist, patient, and health insurance provider, based on costs, rewards, and level of compliance of the patient with the dentist and the health insurance provider.

These basic psychosocial and social interactional models are useful in examining how individuals make decisions in regard to visiting a dentist, and they have made important contributions in clarifying the utilization of health services. However, these traditional health belief models have notable limitations. First, they are biased in their emphasis on "rationality" – that is, patients are assumed to act as rational agents capable of conceiving a symptom as a threat, and medical professionals are assumed to have the ability to reduce this threat. Empirical studies have revealed only weak relationships between psychological concepts such as motivations, beliefs, attitudes, and opinions with preventive dental health behaviors, particularly among vulnerable populations such as minority patients and children in poverty [56,57].

Moreover, the health behavior models employ a very narrow focus on individual psychological states and ignore the pivotal role of macro level influences – political economy, wealth distribution, and the unequal distribution and dissemination of new technology – along with the possible interaction of macro and micro level factors. Oral health is clearly situated within the larger framework of political, economic, and cultural forces. Empirical quests to discover the connection among biological processes, social structure, and oral health disparities have generally focused on measurable individual attributes (e.g., socioeconomic background, past experience with dentists, and the perceived ability of the patient to recognize his or her own ill health conditions) and have ignored the larger social and cultural contexts in which individual characteristics are defined.

For example, in epidemiological studies, socioeconomic status, or social class, is almost universally transmuted methodologically into attributes of individuals (e.g., income, occupational status, educational attainment). Socioeconomic status is a multifaceted and historical phenomenon defined and reproduced via social processes and power relations structuring materialistic conditions and life changes over time. Few empirical oral health models have made visible the contextual qualities in which structural/political forces constrain and perpetuate processes of social differentiation in society, which subsequently contribute to oral health disparities.

Fourth, these models fail to incorporate social capital factors, such as a "lay-referral" system, in which individuals share experiences and seek advice on their symptoms from family, friends, or relatives. In vulnerable populations, which often lack direct access to health-care professionals, social networks may have a strong influence that helps individuals seek needed health care [58-60]. For example, a substantial proportion of ethnic minorities live in enclave communities in which a distinct "collective lifestyle" shapes local customs, values, norms, perceptions, and habitual practices (e.g., culturally specific food and personal hygiene practices), and few ethnic dentists are available to provide adequate dental care. As cultural perceptions are intimately tied to perceptions of the dynamic power relations between dentist and patient, ethnic minorities may come to rely mainly on their inherited social referral system to avoid contacts with dentists lacking the cultural competency to understand and appreciate their distinct habitual and dietary practices. Similarly, ethnic minorities with language barriers may rely primarily on their inherited community social networks when seeking dental care to avoid cultural and language barriers.

Fifth, the social interaction models have responded to critiques of the health belief model by placing more emphasis on the reciprocal and interactional nature of the dentist-patient relationship. Empirical support for this model, however, is sparse. Few empirical attempts have incorporated both the health belief and social interactional models in researching oral health disparities, particularly in studying the underlying dimension of social distance in relation to the cause of under-use among vulnerable minority populations.

Finally, most of the published models concentrate on dental care as the major determinant of oral health and oral health disparities. Hay and colleagues, in an application of the Grossman model described above, reported analyses showing that number of dental visits had a negative effect on the number of decayed teeth, demonstrating a beneficial effect of dental care [61]. Number of visits was related positively to oral hygiene but was not significant statistically. These results must be regarded as tentative, however, because of the study's small sample size. Newhouse and Friedlander found no association between the prevalence of periodontal disease and an area's dental resources, measured by the dentist-population ratio, in 39 areas of the U.S. [62]. Weinstein and colleagues found no relationship between time spent with dental hygienists and levels of oral plaque and inflammation [63]. Moreover, education efforts toward patients were inversely related to the level of the problems presented. That is, healthier patients received more attention, and patients with more problems received less attention [64].

Dental care, particularly preventive dental care, is an extremely important determinant of oral health inequality. Less is known about the broader social and cultural determinants that influence oral health practices, the values and beliefs about teeth, mouth, and face, and how these values, beliefs, and practices vary across different social and cultural groups. Dental care and dental insurance are not the major determinants of oral health disparities [65]. Concentrating only on providing access to dental care may detract from the more powerful effects of social stratification, power differentials, and the understanding of different cultures and their beliefs and practices that contribute to oral health. Broadening the scope of the conceptual framework is intended to open the door to new and different channels for intervening, while recognizing that professional care is necessary throughout life to maintain good oral health and to benefit from evidence-based technological advances.

## Toward a Conceptual Framework for Oral Health Disparities

**Figure 1:**
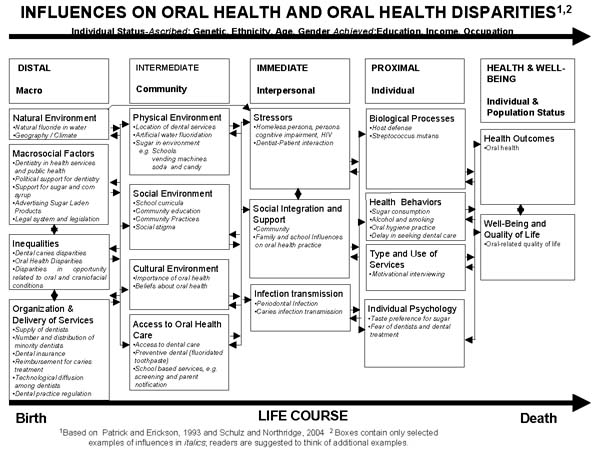
Influences on Oral Health and Oral Health Disparities.

Utilizing models from epidemiology and sociology [66-69], we identified a series of causal mechanisms through which institutional, political, community, and social interaction factors contribute differential impacts on individual oral health and overall quality of life. In Figure 1, titled "Influences on Health and Oral Health Disparities," we propose a comprehensive conceptual framework that encompasses oral health as a dynamic process in which a variety of forces operate both to perpetuate and to reduce social disparities in oral health.

These forces are arrayed according to the approximate time sequence in producing oral health disparities:

• distal/macro level factors (e.g., natural environment, macro-social factors, social inequalities, organizational practices, and delivery of oral health services);

• intermediate/community factors (e.g., environmental forces, social/cultural context, access/utilization of oral health care);

• immediate/interpersonal factors (e.g., negative stressors, social integration, infection transmission);

• proximal/individual factors (e.g., biological processes, personal oral health hygiene practices, psychological state).

At the bottom of the figure, the arrow indicates that the relative contribution of these determinants varies across the life course from birth to death. Underneath the column headings (for example, DISTAL/Macro), the boxes contain selected examples in italics (*Natural fluoride in water*) of different influences. These examples can be added to by readers and researchers to customize the figure to different influences and different disparities.

Our framework outlines the multiple and dynamic pathways through which underlying political, social, cultural, and economic forces influence oral health. Here is a brief description of each.

### Distal/macro factors

At this broadest level, disparities are produced and reproduced by political, economic, social, and cultural forces. For example, in the United States, national policies, legal frameworks, and social ideologies have created and perpetuated unequal distribution of wealth, spatial segregation, and concentrations of poverty. Vulnerable populations, including African Americans, Latinos and new immigrant groups experience limited access to opportunities (e.g., jobs with medical/dental insurance, which affect access to dental care).

For our purposes, macro factors include the allocation of resources to organizations that perpetuate preferential delivery of oral health education and technologies. Private dentists are more likely to practice in urban metropolitan areas with a high proportion of middle- and higher-income residents than in rural areas. Currently, there are no national statistics that describe the distribution of dentists by income areas. Instead, estimates are obtained per state. For example, Krause, Mosca, and Livingston (2003) found that in Mississippi, with a high proportion of rural residents, approximately 40 percent of the licensed dentists practice in only two metropolitan areas – Jackson and the Gulf Coast [70]. As a result, 38 of the 82 counties in Mississippi have 4,000 or more persons per dentist. Mertz and Grumbach (2001) investigated distribution of dentists by geographic areas with low socioeconomic levels in California [71]. Results showed that about 20 percent of California communities had a shortage of dentists. In particular, two-thirds of dentalshortage communities were rural with high percentages of minorities, children, and low-income persons.

Dental insurance is often considered one of the primary links to maintaining oral health. Empirical studies have repeatedly documented lack of access to dental insurance as a factor for widespread dental caries in young children [72,73].

### Intermediate/community factors

These factors include the physical, social, economic and cultural environment of a particular community. Decision makers wield power over services, such as transportation and health care, land use, and access to a healthy environment. At this level, public input can influence city and county councils, school boards, zoning committees, and medical boards. This level becomes important as citizens try to negotiate access to health care, education, transportation, jobs, and social services. In wealthy communities, individuals will likely have political and economic power to influence allocation of resources. Communities with fewer resources may experience greater social, environmental, and psychosocial stressors [74].

### Immediate/interpersonal factors

Immediate/interpersonal factors are the "mediating pathways" within a community. Families, support groups, or other formal or informal networks may intervene to offset distal/macro or intermediate/community factors. For example, a group of parents may ask a school board to set aside a primary grade toothbrushing time after lunch. An environmental group might organize an annual beach cleanup. Influences can be harmful as well. For example, gang violence may cause a community to close a playground or a park. An antifluoridation lobby might work to halt fluoridation plans for a community's water supply.

### Proximal/individual factors

The focus at this level is the actions and beliefs of individuals who make up a community. Personality traits, motivations, values, and personal preferences come into play, along with health needs. Genetic-environmental interaction and organ function, use of community services, and individual psychology are considered. These individual factors influence all other levels. For example, an individual concerned about lack of affordable dental care might encourage neighbors (immediate/interpersonal), his/her city (intermediate/community), and federal agencies (distal/macro) to lobby for local low-cost community dental care or water fluoridation.

Any or all of these levels of social organization can influence dental health and dental care. At the proximal/individual level, a child may become infected with dental caries. Bacterial infection is necessary but not sufficient for developing dental cavities if proper care is available [75]. The child may be deemed eligible for Medicaid (distal/macro factor) and receive care at a local clinic (intermediate/community factor). Health conscious caregivers might schedule the child for regular checkups (immediate/interpersonal factor), and parents might encourage daily tooth cleaning (proximal/individual factor).

In summary, the emphasis in our proposed conceptual framework is on the implications of broad social and economic disparities (macro/distal factors) for the social, environmental, and cultural contexts (intermediate/community factors) that influence interpersonal relationships (immediate/interpersonal factors), and ultimately affect individual quality of oral health and oral-related quality of life (proximal/individual factors).

The varying plausible pathways in our proposed framework (distal/macro, intermediate/community, immediate/interpersonal, and proximal/individual) may exert both uni-directional and bi-directional "push" and "pull" forces on oral health. That is, negative factors at one level may "push off" or "pull in" other levels of factors. Though our graphic presentation may not capture all the interactions within and across varying levels of factors, we hope it will offer an explicit picture to illustrate the complex inter-linkages among macro, meso, and micro phenomena that affect individual oral health.

## Applying the Conceptual Framework: Two Case Studies

To illustrate the utility of our conceptual framework, we use two case studies involving minority populations to trace pathways through which social disparities intersect with social, cultural, and political contexts to affect oral health and overall well-being. Each illustrates the interrelated, overlapping process in which changes at one level induce counter-forces that may mediate/intervene, or perpetuate disparities in oral health [76,77].

### Case Study of Oral Health in Alaska

Alaska Natives, particularly children, are disproportionately affected by oral disease. According to the Oral Health Survey of American Indian and Alaska Native Dental Patients (1999), tooth decay is five times more frequent among Alaska Native children than among the U.S. average population of children 2–4 years of age [77]. Seventy-nine percent of Alaska Native children aged 2–5 years have tooth decay; 60 percent have severe early childhood caries. Further, the prevalence of tooth decay increases with a child's age. Approximately 99 percent of Alaska Native youth 15–19 years old have at least one decayed tooth.

The most frequently cited significant factors for dental caries in Alaska are linked to distal/macro levels – economic change, social disparities, and environmental isolation – that impact the intermediate/community factor of access to professional dental care. Distal/macro factors also include the natural environment, geographic isolation, and an unprecedented economic conversion to a market economy (which directly impacts dietary and cultural practices).

As early as the beginning of the 20th century, Alaska Natives traded furs and other goods for imports of tea, flour, sugar, tobacco, ammunition, and other products that would eventually change levels of self-sufficiency and transform local domestic economies. For the most part, communities provided adequate diets for families with foods taken directly from the local environment, either from the land or the sea. Following World War II, however, the flow of goods, imported first via barge and boat and eventually by airplane, began to affect the relative independence of rural villages.

By the 1970s and 1980s, dramatic increases in the consumption of non-native foods had occurred. Such consumption depended on a shift from subsistence-oriented domestic economies to a wage-earning, mixed market-subsistence economy common to the rest of the United States. This shift was accompanied by increased dependence on subsidies, and with these linked dependencies came an increased flow of material goods into the local communities – especially such items as commercially prepared and packaged foods, coupled with an already prevalent use of the imported foods mentioned above, and an increased dependence on imported fuel. Diets once devoid of all but locally available meats, fish, eggs, greens, and berries suddenly were rich in carbohydrates and sugars, which exposed children to a higher susceptibility to tooth decay [78]. Practices designed to deal with maintenance of oral health as the result of this major diet shift simply did not exist. Traditionally, the occasional "bad tooth" was treated by a local person adept at pulling teeth. Dentistry as practiced elsewhere was unknown.

The problems persist today, compounded by the relative geographical isolation of tribal populations in Alaska (distal/macro factors). It is difficult to attract dentists to remote areas on more than a peripatetic basis and even more difficult in extremely remote rural areas of Alaska to establish sufficient tribal health facilities to provide regularly available treatment (intermediate/community factors). This is true despite intense recruitment efforts and offers of significant financial incentives to attract dentists to Alaska's rural areas. According to a survey in 1999, Alaska tribal facilities have approximately a 25-percent vacancy rate of dentists and a 30 -percent average annual turnover rate [79]. The ratio of dentists to population is 1 to 2,800, compared to 1 to 1,500 individuals in the general population of Alaska [79,80]. The ratios understate the problem, because many communities are geographically isolated, and dentists are concentrated in regional centers, such as Nome, Bethel, Fairbanks, and Kotzebue, often hundreds of miles away from the rural communities they serve.

The Native Health Service has worked hard to overcome the limitations of episodic and symptomatic care and to institute preventive measures, such as fluoridation. Unfortunately, attempts to fluoridate rural water systems are fraught with problems, and capable engineering and control staff are rarely available. In May 1992, an outbreak of acute fluoride poisoning that resulted from inadequate inspection of a water system affected almost 300 people [80]. Thus, fluoridation of central water systems became impossible. Topical fluoride programs have been instituted but are limited by the absence of trained staff in many locales and the relative ineffectiveness of fluoride alone as a preventive strategy in the face of rampant tooth decay, carbohydrate-rich diets, and lack of oral hygiene. Even the cost of fluoridated toothpaste, if it is available in village stores, is double the price in the lower 48.

To address the disproportionately high rate of dental caries in young children in Alaska, the Northwest/Alaska Center to Reduce Oral Health Disparities of the University of Washington launched the *Caries Transmission Prevention in Alaska Native Infants Study *(CTP) to investigate difficulties tied to previous studies of high prevalence of dental caries in most communities [81]. The study goal was to determine if serial use of chlorhexidine mouth rinse and xylitol chewing gum would reduce the vertical transmission of caries between 250 Alaska Native mothers and their infants.

The study protocol involved giving chlorhexidine rinses (alcohol free and previously shown to have an acceptable taste to Native Alaskans) and chewing gum containing xylitol to pregnant female participants in conjunction with a program that would determine participant satisfaction with and efficacy of xylitol/chlorhexidine use. Oral chlorhexidine is a widely available antimicrobial. Xylitol is a naturally occurring sugar available in the United States and elsewhere in chewing gum and foods for diabetics [82]. It is used widely as a sweetening substitute for sucrose and fructose in Northern Europe because of its preventive effect on dental caries[83]. Yet even with strong empirical evidence of xylitol's preventive benefits, this technology is just beginning to be incorporated in dentistry in the U.S. Participants were interviewed throughout various monitoring time points regarding their perceptions of xylitol use, dental caries, and preventive strategies to fight dental caries in young infants.

Early into the study, however, significant problems of subject recruitment and attrition affected the collection of data, and ultimately the study was abandoned [81]. The study also had been plagued by persistent problems in recruiting and maintaining local staff in the remote areas. In spite of their previous experience in Alaska and the enthusiastic acceptance of the program by health providers, the UW research team determined that some of the problems experienced may have been caused by researchers' unfamiliarity with local cultural traditions regarding concerns about chewing gum during pregnancy. One particular cultural barrier appears to have been a fear that chewing gum might harm unborn infants, and several villages declined to allow the researchers to distribute xylitol gum to their pregnant women. Also, the women themselves often felt they could not make the decision to participate without discussion with other family members. When they were approached at a regional center where they went for delivery, the extended family was not available for consultation, so researchers were once again stymied.

Although the researchers made a concerted effort to provide education and health care to potential participants, problems of communication manifested in an inability to establish trust in the villages. A more general lack of understanding of cultural differences by both researchers and potential recruits brought a final halt to the study. As a result, it was not possible to determine whether the chlorhexidine/xylitol gum would have contributed to a reduction in the high level of dental caries that afflicts Alaska Native infants [81]. Dental caries among young infants remains a primary health problem of Alaskan children.

To reduce oral health disparities in Alaska Native populations, oral health promotion programs will need to address cultural differences and collaborate more closely with communities to initiate acceptable education and intervention programs. Programs will need to move beyond cultural and social barriers to implement culturally sensitive care at the immediate/interpersonal level and to eliminate the current gap between dental care professionals and patients. These efforts assume that researchers can learn culturally-aware listening behaviors, openness to culturally inclusive ways to introduce possible oral health promotion and intervention programs to vulnerable communities, and culturally appropriate methods for alleviating community-level reservations about seeking dental care.

High on the list of appropriate behavioral changes is the necessity for researchers to resist attempting to impose "western ideas" on local communities. For example, researchers must set aside the habit of speaking quickly and without regard for those whose habits of conversing may include lengthy pauses before answering questions. To gain long-lasting, trusting relationships, researchers will need to establish rapport both with local tribal leaders and with community members in face-to-face encounters, at the immediate/interpersonal and proximal/individual levels. A long-term horizon is needed.

A new study is underway to investigate difficulties associated with the earlier project, to identify and explore underlying causes of the difficulties, and to generate hypotheses for an alternative and more culturally sensitive model [84]. The primary goal is to use ethnographic [qualitative] methods and approaches common to socio-cultural research to reinvestigate the Alaska Native community perceptions of oral health, the priority placed on infant/child oral health, and the fear levels associated with oral health in general, and with the participation of recruited subjects in medical/dental research interventions in particular. Clearly, rapport, trust, and tribal cooperation/collaboration and endorsement are necessary to address community oral health issues.

Using the conceptual framework as applied to this case study, social integration and cultural in-competency, in combination with distal/macro and intermediate/community level factors, "pull-in" increasingly negative effects of social and economic disparities in oral health. That is, while the Alaska Native population faces the on-going negative effects of economic and social changes, geographic isolation continues to contribute to lack of access to oral health professionals (*distal/macro factors*→*intermediate/community factors*), and at the same time, social opposition against the study (*intermediate/community factor*) contributes to its failure. Social opposition was partially related to a lack of cultural competency of the researchers, who were unfamiliar with the local circumstances of each participant community and unable to address concerns over traditional tribal beliefs about gum chewing during pregnancy and other concerns growing out of the traditional cultures (immediate/interpersonal factor). Until an effective health intervention program addresses the distal/macro and immediate/interpersonal barriers, economic, social, and environmental disparities will continue to negatively impact the population.

### Oral Health Among the Latino Population

Latinos, the nation's largest minority group, have the highest rate of untreated tooth decay and the lowest level of dental visits of all racial and ethnic groups in the United States [85]. According to 1988–1991 data from the Third National Health and Nutrition Examination (NHANES III), Latino children receive few preventive services; for example, only 10 percent of 8-year-old Mexican-American children received sealants, compared to 29 percent of non-Hispanic white children [86]. Mexican-American adults experience tooth decay disproportionately as well. The percent of untreated oral disease for Mexican-American adults was 40 percent in 1991, compared to only 24 percent for non-Hispanic whites [87]. According to the National Health Interview Survey (1999), Mexican Americans, Cuban-Americans, and Puerto Ricans visited the dentist less frequently than did non-Hispanic whites. In particular, Hispanic children of all ages are twice as likely to have untreated dental caries in their permanent teeth as non-Hispanic white children [88].

The most frequently cited barriers to oral health care among Latinos are related to distal/macro and intermediate/community factors. Disadvantaged economic positions and immigration contribute to differential access to oral health. Latino families experience substantial barriers to receiving dental care, including lack of dental insurance (distal/macro factor), under-representation of Latinos in the U.S. dental work force (distal/macro level factor), and cultural and linguistic obstacles (intermediate/community factors). For example, studies of the effects of acculturation on oral health among Latino populations consistently show that acculturation is a predictor of better oral health, increased utilization of oral health services, and more positive self-rated oral health [89-91]. In studies, Latinos frequently cited language barriers as a deterrent to effective interaction between themselves and health care providers. Latinos who speak primarily English at home were more likely to use dental health services than those who speak primarily Spanish at home [92]. Poor English skills create substantial difficulties and fears for Latinos, making them less willing and trusting toward dental care professionals. In short, due to unequal distribution of wealth, lack of access to health-care insurance (distal/macro factor), cultural barriers (intermediate/community factor), and a language barrier (proximal/individual factor), Latinos are less likely to make regular visits to dentists, or to attend to tooth decay. Some Latinos may also fear that using public health programs could expose them to harassment by immigration authorities (distal/macro factor). One of our researchers (PM) conducted a preventive dental screening and topical fluoride program in a church in rural Washington State only to be told afterward that the federal government's Immigration and Naturalization Service swooped down on the population served as they left the church hall.

To reduce oral health disparities among Latinos, oral health intervention programs will need to develop cultural and language intervention programs at the intermediate/community level. At that level, oral health interventionists can begin to build culturally competent dental homes, which offer culturally relevant, specific dental health information for new immigrants and young children. Latino patients, with a low level of English language skills and high level of fear, need to interact with Spanish-speaking, culturally competent health care professionals. Dental outreach clinics may coordinate efforts with local community centers to deliver services to new immigrants who lack access to dental insurance. In particular, oral health information in Spanish is needed to reach recent immigrants who fear dentists and health care professionals. Emphasis should be on oral communication (in Spanish) instead of written health information.

## Implications for a New Direction

The structure of our conceptual model has explicated the process in which environment, economy, social context, cultural practices, social integration, individual factors, and biological factors influence oral health. Our model has incorporated aspects of the traditional health belief model and the social interaction model. Within the context of the traditional health belief model, we recognize that one's health beliefs reflect a process of rational decision making, based on the severity of the oral illness, past experience with dentists, and the potential costs and rewards of a dental visit. In relation to the social interaction model, we acknowledge that an episode of dental care is an on-going interactional process in which dentist, patient, and oral health service payer all influence the nature of the social exchange.

We have responded to critiques of both the traditional health belief model and the social interaction model by combining both models and incorporating a new aspect in which we emphasize the interrelated dynamic nature in which social processes both influence and are influenced by various aspects of factors embedded in our model. Cultural and social processes may directly influence community norms and individual behaviours. In terms of dental care, a dental visit requires not only a patient's self recognition of a dental illness, but also social exchange between dentist and patient. The main contribution of our conceptual model is that we emphasize both the "pull" and "push" forces of each of the social processes in which social inequalities are produced and reproduced.

Our case study of the Alaska Native population has elucidated how previous experience and one's health beliefs are conditioned by one's immediate relations to family, community, and to the overall structural forces of society. In addition, we have discussed structural factors as a significant influence on oral health. For example, the most crucial problems in the Alaska Native population are related to the geographic isolation of communities and their cultural practices. Thus, given that this geographic isolation is unlikely to be altered in the near future, oral health workers will need to redouble their efforts to understand Alaska Native cultures and to develop culturally sensitive and appropriate efforts to target the disproportional spread of oral health disease among young children. Similarly, our case study on Latinos pointed to structural and immediate factors – limited access to dental health insurance, cultural and language barriers, and fear – as the primary forces leading to high rates of oral health disease.

Both studies lead us to conclude that oral health interventions must begin to permeate all levels – distal/macro, intermediate/community, immediate/interpersonal, and proximal/individual – in order to induce effective strategies for the elimination of disparities in oral health. The following section proposes interventions at each level.

## Interventions at Each Level

### Distal/Macro level

Current inequalities in oral health (for example, differential access to oral health care and discriminative practices of the health care service providers) must be redressed at the distal/macro level if the U.S. is to move toward equality in oral health care.

Nationally, political parties and lobbying efforts dictate distribution of resources. Social groups with more resources are better equipped to negotiate and bargain for goods and services than social groups with fewer resources. Minorities or recent immigrants are less able to use their political rights to activate structural and political support to fight violent crime, ensure medical and dental services, and ensure human rights. Moreover, social groups with fewer resources may be less likely to question the unequal distribution of wealth and power, thereby increasing their vulnerability to discrimination and unequal treatment.

In our opinion, to combat disparities in oral health and other health and social disparities, we need a "structural readjustment" in the political process that allows all members, regardless of social status, equitable access to political power to achieve equal access.

Oral health is intimately tied to national (distal/macro) policies. Policies that promote opportunities for the lower class may also reduce health risks and financial barriers to dental care [25]. We cannot adjust for equitable access to dental care without addressing the political and economic processes that cause poverty and disparities in oral health. Financing for better education and installation of family wage policies are fundamental to improving opportunities for all.

Programs that reduce discriminative practices and increase multicultural awareness are crucial to a stable economy in which all social groups may benefit. Discriminative practices that permeate the dental setting will require effort from medical and dental associations in order to bring forth effective measures. For example: *Eliminate discriminative practices of dental professionals: *Dentistry literature has consistently indicated negative attitudes towards poor people. Oral health intervention efforts must address this cultural gap by improving dentists' awareness of minority cultural values and practices. Multicultural studies could be incorporated into the dental school curriculum and students could begin to work with skilled medical interpreters before graduation. The American Dental Association could organize conferences to address multicultural issues in order to narrow the cultural gap between dentists and patients.

#### Improve financing for Medicaid patients and dentists

One of the most important causes of under-use of Medicaid has been its rejection by dentists because of low fees and inefficient reimbursement systems. Yet, often the professional associations themselves have failed to place better funding for Medicaid at the top of their political agendas instead claiming their political action committees are impotent to change state-based policies. The net result is that low-income patients, even with Medicaid, are unable to find dental care. These structural barriers can be reduced by increasing Medicaid reimbursement fees, improving the efficiency of payment schedules, expanding the types of dental treatment options, and encouraging dentists to participate in Medicaid programs. The will to find the opportunity for these structural changes is required.

#### Expand Public Health programs

Public health programs are an important vehicle for improving oral health among the poor. These may include expanding fluoridation programs in poor neighborhoods and expanding the use of dental health care teams. Fluoridation benefits everyone who drinks from the water supply irrespective of their own resources or behavior, which means that socioeconomic gradients in oral disease are blocked and everyone benefits equally [93,94]. In New Zealand and many other countries, dental therapists have been trained to provide comprehensive primary care to school children [72]. The training curriculum for New Zealand dental therapists consists of two academic years, both of which are 32 weeks long. These therapists provide a full range of care for children in school-based clinics. The Alaska Native Tribal Health Consortium, under the provisions of Native sovereignty, has deployed an initial group of New Zealand-trained therapists in rural villages. The American and Alaska Dental Associations are vigorously opposing this move. Efforts to establish a training program and further deployment in the U.S. also have incurred strong resistance, including lawsuits and attempts to get Congress to ban the practice, even though the therapists are trained to work in collaboration with dentists, and the integrated system in which they all work allows for referral of problems to dentists and specialists in regional centers or to the Alaska Native Medical Center in Anchorage.

Training programs for dental hygienists in the United States also lag far behind the types of programs in other countries for dental therapists, and the limited independent practice of hygiene lacks integration into a coherent system. Nevertheless, with proper training, a new focus, and deployment to underserved areas, dental hygienists might play a role in reducing inequities. The organized dental profession opposes such practices because the current system of deployment of dental hygienists as employed production workers in affluent private practices is highly profitable.

The bottom line for reducing oral health disparities through dental care is likely to be no different from the challenge for health disparities in general. Many European nations have incorporated health care as a centralized national policy, in which all members receive equal access to care, including dental care. As one of the wealthiest countries in the world, why cannot the U.S. also afford to adopt a centralized system of health care?

### Intermediate/Community Level

At the intermediate/community level, health insurance is generally delivered as part of employment benefits. However, many low wage earners are unable to receive dental or medical insurance due to low number of work hours. The distribution of health care is intimately tied to the distribution of employment opportunities. In the public sector, employment decisions are based on bureaucratic procedures that account only for applicants' skills and qualifications, irrespective of gender and race. However, in the private sector, hiring practices may include reliance on lay referral systems, such as internal networks. Discriminative practices may include use of referral systems that exclude members of certain social groups based on stereotypes. To reduce oral health disparities at the intermediate/community level, we need to first examine the differences in hiring practices and evaluate how these processes may be altered.

#### Alternative delivery systems

The current oral health delivery system is bifurcated. Patients with medical and dental insurance seek treatment at private clinics; patients with Medicaid attend public health clinics. (Most are turned away from private dental offices.) To adjust the bifurcated system to a more dynamic system, dentistry must allow for alternative delivery systems, such as retail dentistry, mobile dentistry, independent practice of dentist hygienists, and deployment of dental therapists.

#### Expand current systems

Access to dental care for the poor can also be in increased by expanding the number of public dental clinics and primary care programs in the community [69]. At community dental clinics or community dental homes, children and low-income parents can request and receive information and education about the importance of oral health, which in turn can help instill good oral health hygiene habits. Furthermore, community health clinics are staffed by local community members, who are equipped to understand and comprehend distinct cultural practices in the community and to aid in reducing the cultural gap between dentists and patients. In ethnic enclave community clinics, new immigrants may also rely on translators for help in communicating with dentists and other health care professionals.

#### Community collaboration and mobilization

Community-organized efforts are intimately tied to how resources are allocated in society. Citizens can influence zoning policies and can tap into resources to build good schools, recreational facilities, parks, and community centers. That is, community-organized efforts can "push back" forces that contribute to unequal access and subsequently induce change at the proximal/individual level. For example, a community may demand changes to zoning policies in order to promote financial investments such as a business district, supermarkets, and banks. Access to a healthy neighborhood can decrease residents' personal stress levels by providing such amenities as fresh produce, safety, and good schools.

Community efforts can begin to improve oral health via coordination with already existing school and community programs, such as Parent Teacher Student Associations and local community centers. Promotional and prevention efforts may be incorporated into school curricula, concentrating on good oral hygiene via school- and community-based programs. These programs can improve social integration and encourage parents, teachers, nurses, and community leaders to facilitate, educate, and share health information.

Community information campaigns that stress health literacy can bring attention to the problems of untreated tooth decay among the poor. Low-income wage earners, who lack access to dental insurance, need to receive information regarding the benefits of Medicaid and of attending to their health care needs. Patients from disadvantaged social groups often want to have information regarding their health status [95]. They often need to know how to recognize signs of tooth decay in young children. This important oral health information may come from community health care professionals who already have a close relationship with local residents, and who may also be able to eliminate language barriers. In areas that lack community health care professionals, new programs must be developed to address the problem of lack of access to oral health information.

### Proximal/Individual Level

Health disparity interventions at the proximal/individual level, when carefully applied, can reduce stressors and oral health disparities. Individual intervention efforts may include the following:

-Learning to practice oral health hygiene to help fight bacteria and maintain good oral health.

-Visiting dental offices or dental clinics and then transferring newly-learned knowledge to family and friends.

-Introducing fresh produce and nutritional supplements into the family diet and limiting sugar intake.

## Action at All Levels

The underlying themes for interventions at all levels are financial support, structural change, conscious effort, and education. All involve conscious effort. That is, successful interventions and policy efforts must incorporate a 'fundemental-social-cause approach' with contextually population based health interventions that automatically benefit everyone, irrespective of their socio-economic status, resources, or behaviors. In the United States, interventions must be organized and priortize to people at all socioeconomic levels, with a specific target to address the special needs of resource poor groups who may face obstacles and barriers in implementing health interventions. Hence, we need to promote policies that promote the elucidation and elimination of SES gradient across population groups – via increases of the socioeconomic resources available to resource poor groups [93]. The following recommendations recommend action at all levels:

### Financial support

Dentistry needs financial resources to expand delivery services, improve Medicaid reimbursement, increase education, and reduce poverty at the societal level. These interventions all require efforts by federal and state governments to launch new social policies and allocate funds to reduce social disparities (which contribute to oral health disparities).

### Structural changes

Only when structural problems, such as the political process in which social groups coordinate efforts with interest and lobbying groups, are fundamentally altered, can poverty be mitigated. It is unlikely that such structural readjustment will be realized in the near future. Interventions may yet develop to improve the Medicaid insurance program. Expansion of dental health facilities and Medicaid insurance for vulnerable populations, are important components of oral health. Patients from disadvantaged backgrounds may need to be able to find dentists willing to accept Medicaid insurance and treat their dental disease. Interventions to increase dentists' participation in Medicaid are basic to increasing and improving care to its covered population. Programs to motivate dentists to treat Medicaid patients, such as the ABCD program in Washington State, have demonstrated success at increasing the number of Medicaid providers.

Children living in poverty are likely to have limited access to dental care. Improvement of Medicaid insurance programs with acceptable reimbursement rates may encourage more dentists to accept patients with Medicaid insurance. Studies have shown that increased reimbursements increase the number of patients per dentist, but these increases can also bring new dentists into the Medicaid field [10]. Educational programs designed to increase the skills of dentists to effectively treat children are needed.

### New Technologies

Dental professionals are important agents who directly influence the prevalence of dental caries. They are also the crucial link between newly invented technology and its distribution to patients who need it. New technologies such as fluoride varnish and xylitol toothpaste are low-cost, effective preventive agents against dental caries. Yet, despite their documented effectiveness, they remain underused in clinical practice in the United States. In a recent study among general dentists on the use of fluoride varnish in adults [96], only 44 percent were found to use fluoride varnish regularly on their adult patients. Many dentists cited the lack of awareness, lack of convincing evidence of favorable benefit to cost, patients' lack of knowledge regarding fluoride varnish, and lack of acceptance among patients as reasons for the low rate of use. Clearly, evidence supports expansion of the dentist's role to include educating patients and disseminating proven and effective technologies for preventing dental caries.

### Turning resistance into cooperation

Efforts to address social disparities in children's oral care must anticipate resistance from political interest groups, the private sector, and organized medical professional societies. For example, distribution of fluoride varnish and xylitol gum or candies by school nurses may be opposed by professional societies as interfering with the rights of dental professionals, who currently are the only legal recourse for treatment of dental caries.

Meanwhile, local communities, concerned medical professionals, non-profit social policy groups, and individuals may organize to combat the opposition by increasing awareness of oral health in society. By involving themselves in distribution of societal and political resources, including community planning, school curricula, and improved access to health care, concerned individuals and dental professionals will have a chance to carefully elucidate and effectively eliminate underlying factors that cause and drive social disparities in oral health.

## Competing interests

The author(s) declare that they have no competing interests.

## Authors' contributions

All of the authors contributed to this review.
